# Antitumor and antimetastatic activities of a novel benzothiazole-2-thiol derivative in a murine model of breast cancer

**DOI:** 10.18632/oncotarget.14431

**Published:** 2017-01-02

**Authors:** XiaoLin Hu, Sen Li, Yan He, Ping Ai, Shaoyong Wu, Yonglin Su, Xiaolin Li, Lei Cai, Xingchen Peng

**Affiliations:** ^1^ Department of Nursing, West China Hospital, Sichuan University, Chengdu, China; ^2^ Department of Spinal Surgery, Traditional Chinese Medicine Hospital of SouthWest Medical University, Luzhou, China; ^3^ Department of Medical Oncology, Cancer Center, State Key Laboratory of Biotherapy, West China Hospital, Sichuan University, Chengdu, China; ^4^ Department of Rehabilitation, West China Hospital, Sichuan University, Chengdu, China; ^5^ Department of Pathophysiology, Basic Medical College, Jilin University, Changchun, China; ^6^ Hepatobiliary Surgery Institute, Southwest Hospital, Third Military Medical University, Chongqing, China

**Keywords:** benzothiazole-2-thiol derivative, breast cancer, proliferation, metastasis, apoptosis

## Abstract

The prognosis of metastatic breast cancer is always very poor. Thus, it is urgent to develop novel drugs with less toxicity against metastatic breast cancer. A new drug (XC-591) derived from benzothiazole-2-thiol was designed and synthesized in our lab. In this study, we tried to assess effects of XC-591 treatment on primary breast cancer and pulmonary metastasis in 4T1 mice model. Furthermore, we tried to discover its possible molecular mechanism of action. MTT experiment showed XC-591 had significant anti-cancer activity on diverse cancer cells. Furthermore, XC-591 significantly suppressed the proliferation of 4T1 cells by colony formation assay. The *in vivo* results displayed that XC-591 could inhibit the growth and metastasis in 4T1 model. Moreover, histological analysis revealed that XC-591 treatment increased apoptosis, inhibited proliferation and angiogenesis *in vivo*. In addition, XC-591 did not contribute to obvious drug associated toxicity during the whole study. Molecular mechanism showed XC-591 could inhibit RhoGDI, activate caspase-3 and decrease phosphorylated Akt. The present data may be important to further explore this kind of new small-molecule inhibitor.

## INTRODUCTION

Breast cancer incidence and mortality have been increasing in China. It has been estimated that 268,600 Chinese women developed breast cancer and 69,500 died of breast cancer in the year 2015 [1]. Therapeutic options such as chemotherapy, radiotherapy, hormonal therapy, and targeted therapies are often used to treat patients with breast cancer [2]. In the last decades, early detection and proper treatment have helped reduce breast cancer mortality. However, distant metastases are still the main cause of breast cancer associated deaths [3]. Thus, it is urgent to develop novel drugs with less toxicity against metastatic breast cancer. Considering safety and increasing anti-cancer efficacy, small-molecule inhibitors are being deeply investigated as a new treatment method.

Benzothiazole derivatives have diverse biological functions, including anti-inflammatory, anticonvulsant, antimicrobial and antitumor activities [4, 5]. But previous studies used 2-aminobenzothiazoles or 2-arylbenzothiazoles to design benzothiazole derivatives. In our lab, we used the benzothiazole-2-thiol as a functional group to develop a new series of compounds. Among these small-molecule compounds, XC-591 had obvious anti-tumor activity on diverse cancer cells *in vitro*, which was more effective than cisplatin (a platinum based chemotherapeutic drug) [6]. However, the exact anti-cancer effects of XC-591 *in vivo* have not been reported. In this study, we attempted to investigate the effect of XC-591 on suppressing both murine breast cancer and pulmonary metastases. In addition, possible molecular mechanism of action was also studied.

## RESULTS

### RhoGDI was increased in cancer cells

**Figure 1:**
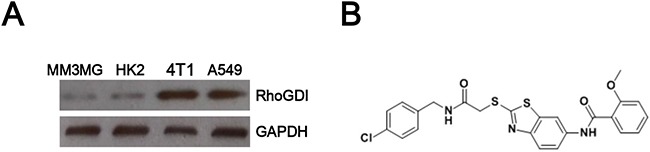
**A**. RhoGDI expression was increased in cancer cells. **B**. The chemical structure of XC-591.

Western blot was conducted to check RhoGDI expression in cancer cell lines (including 4T1 and A549) and normal cell lines (including MM3MG and HK2). RhoGDI was obviously increased in two cancer cell lines, when compared with two normal cells (Figure 1A).

### The cytotoxicity effect of XC-591 on tumor cells

RhoGDI specific inhibitor XC-591 was synthesized in our lab and the chemical structure was shown in Figure 1B. As shown in Table 1, XC-591 inhibited proliferation of many cancer cells (including CT26, 4T1, LLC, B16, MethA, A549, HeLa and DU145). Howerver, no apparently toxicity on normal cells (including MM3MG, HK2 and LO2) was observed (Table 1). Compared with other tumor cells, 4T1 was more sensitive to XC-591 than others and thus was selected for further study.

**Table 1:** The cytotoxicity effect of XC-591 on tumor cells and normal cells

Cell line	Cell type	IC50 (μM)(mean±S.D.)
CT26	Mouse colon carcinoma cell line	4.3±0.8
4T1	Mouse mammary tumor cell line	1.2±0.3
LLC	Mouse Lewis lung carcinoma cell line	6.5±0.4
B16	Mouse melanoma cell line	5.6±0.9
MethA	Mouse fibrosarcoma cell line	12.3±2.8
A549	Human lung carcinoma cell line	2.4±0.7
HeLa	Human cervical cancer cell line	4.3±1.6
DU145	Human prostate cancer cell line	1.9±1.1
MM3MG	Mouse mammary normal epithelial cell line	>40
HK2	Human normal kidney cell line	>40
LO2	Human normal liver cell line	>40

Each cell line was treated with XC-591 for 48 h. Cell viability was detected by MTT assay. Data are expressed as the mean±S.D. from three independent experiments.

### XC-591 suppressed proliferation of 4T1

**Figure 2:**
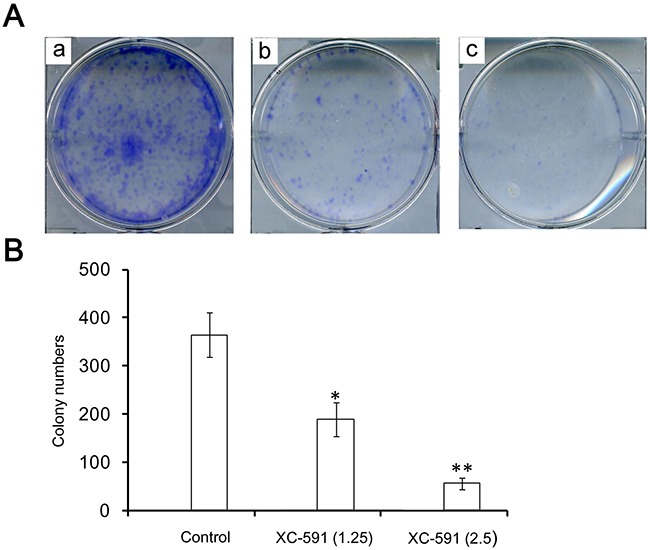
XC-591 could inhibit proliferation of 4T1 *in vitro* **A**. Representative pictures from colony formation assays. a: untreated group; b: 1.25 μM XC-591 treated group; c: 2.5 μM XC-591 treated group. **B**. Colony quantification of 4T1 cells treated with 0, 1.25 and 2.5μM of XC-591, respectively. * indicates P < 0.05, ** indicates P < 0.01.

Colony formation assay demonstrated that there was a marked decrease in the colony number from 364±46.4 in the untreated 4T1 cells to 189±35.2 and 56±12, respectively, in 1.25 and 2.5 μM XC-591 treated tumor cells (P<0.05) (Figure 2). These results showed that the proliferative activity of 4T1 cells was inhibited by XC-591 in a dose-dependent way.

### Anti-tumor effect of XC-591 *in vivo*

**Figure 3:**
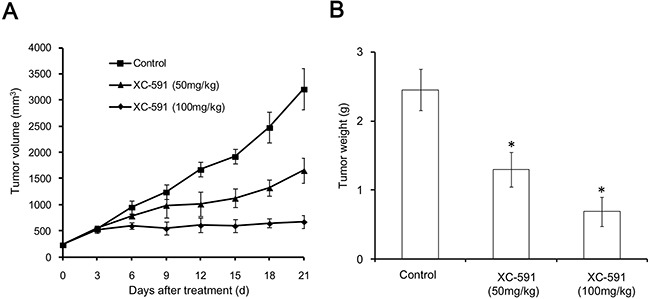
Anti-tumor effect of XC-591 *in vivo* **A**. Tumor growth curves. **B**. Tumor weight. * indicates P < 0.05.

As shown in Figure 3A, it is obvious that the treatment with XC-591 resulted in primary tumor growth regression of 48.5% and 79%, in 50 mg/kg XC-591 treated group and 100 mg/kg XC-591 treated group respectively, when compared with the untreated group. The similar results about tumor weight were also observed. We found the average weight of the tumors was decreased by 46.9% and 71.8% in 50 mg/kg XC-591 treated group and 100 mg/kg XC-591 treated group, respectively, when compared with the untreated control (Figure 3B).

### XC-591 inhibited the metastasis of 4T1 from the primary site to lungs *in vivo*

**Figure 4:**
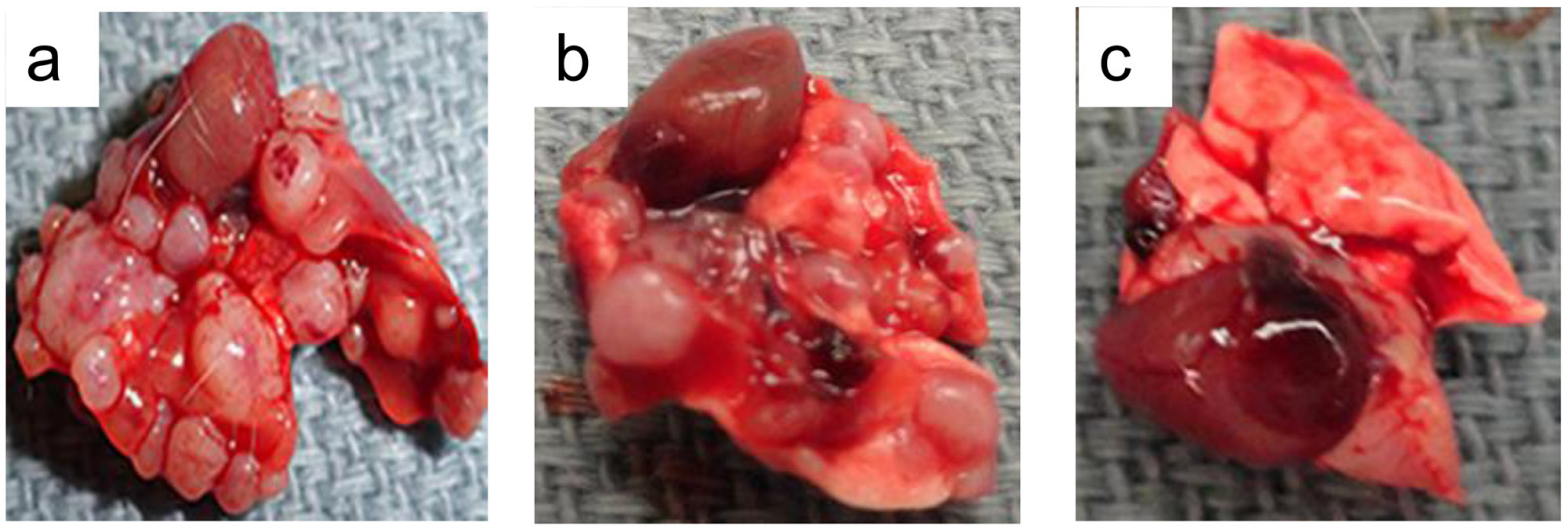
Representative pictures from metastatic lungs in mouse 4T1 model **a**: untreated group; **b**: 50 mg/kg XC-591 treated group; **c**: 100 mg/kg XC-591 treated group.

As shown in Figure 4 and Table 2, our data showed that the untreated group had a median of 40.5 metastases/lungs (range, 28-56 metastases). However, the median of 50 mg/kg XC-591 treated group was only 19.5 metastases/lungs (range, 13–27 metastases). Furthermore, mice treated with 100 mg/kg XC-591 had a median of 6 metastases/lungs (range, 0–8 metastases). More importantly, 3 mice with no visible lung metastases were observed in the group treated with 100 mg/kg XC-591 (Table 2). Thus, XC-591 could inhibit primary tumor growth and distant metastases in 4T1 model.

**Table 2:** Lung metastatic nodules of each group

Groups	Median no. of metastases (range)/lung	% metastasis-free mice
Control	40.5 (28-56)	0
XC-591(50mg/kg)	19.5 (13-27) *	0*
XC-591(100mg/kg)	6 (0-8)*	30*

*significantly (P < 0.05) differs from the control groups according to Mann-Whitney test.

### Inhibition of proliferation (PCNA) and increase of apoptosis (TUNEL)

**Figure 5:**
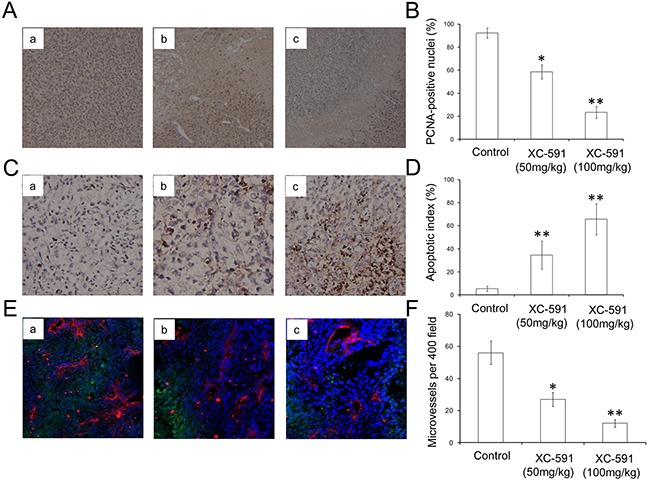
PCNA immunohistochemistry, TUNEL analysis and CD31 staining **A**. Representative pictures from PCNA immunohistochemistry. a: untreated group; b: 50 mg/kg XC-591 treated group; c: 100 mg/kg XC-591 treated group. **B**. Quantified values shown were the average percentage of PCNA-positive nuclei. **C**. Representative pictures from TUNEL. a: untreated group; b: 50 mg/kg XC-591 treated group; c: 100 mg/kg XC-591 treated group. **D**. Percent apoptosis in each group. **E**. Shown are representative sections from CD31 staining. a: untreated group; b: 50 mg/kg XC-591 treated group; c: 100 mg/kg XC-591 treated group. **F**. Quantified values shown were MVD in each group. * indicates P < 0.05, ** indicates P < 0.01.

Tumor sections of each group were stained with anti-PCNA antibody and TUNEL reagent in order to evaluate proliferation and apoptosis rate. Compared with the control group, XC-591 significantly decreased percentages of PCNA-positive nuclei (Figure 5A&5B). Meanwhile we could find a large area of necrosis in the sections of both XC-591 treated groups (Figure 5A). Moreover, in a concentration-dependent way, XC-591 increased percentage of TUNEL-positive nuclei, when compared with the untreated group (Figure 5C&5D). Thus, XC-591 could directly inhibit proliferation and induce apoptosis *in vivo*.

### XC-591 inhibited angiogenesis *in vivo*

As shown in Figure 5E&5F, compared with the untreated group with high MVD, lower MVD could be observed in XC-591 treated groups. Thus, these data showed that XC-591 could inhibit angiogenesis.

### Toxicity assessment

**Figure 6:**
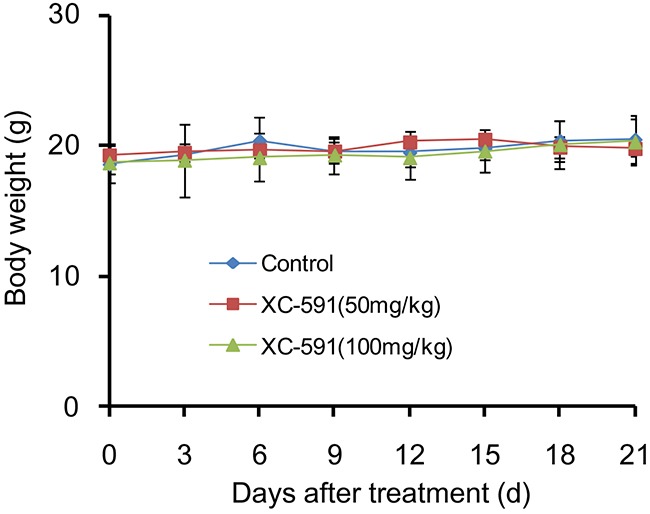
Body weight curves of each group There were no significant differences in body weight among the three groups (P>0.05).

To assess the possible adverse effects of XC-591, weight of mice was monitored every 3 days during the experiment. No weight changes were found after XC-591 treatment (Figure 6). In addition, no ruffled fur or toxic death was observed in the XC-591 treated groups. Furthermore, as shown in Table 3, XC-591 treatment did not contribute to blood toxicity and liver toxicity (P>0.05, respectively).

**Table 3:** The blood toxicity of XC-591 treatment

Treatment	RBC (×1012/L)	HGB (g/L)	WBC (×109/L)	PLT (×109/L)	ALT (U/L)	AST(U/L)
Control	7.6±0.3	138.2±13.6	7.3±0.5	543.7±18.9	32.6±2.3	98.6±6.8
XC-591(50mg/kg)	7.7±0.7	136.1±17.3	7.6±0.8	523.7±16.7	36.4±4.5	96.3±12.3
XC-591(100mg/kg)	7.5±0.5	141.2±15.4	7.5±0.9	537.9±21.5	35.3±3.5	96.4±6.4

Data are expressed as the mean±S.D.

### XC-591 inhibited RhoGDI, activated caspase-3 and reduced phosphorylated Akt

**Figure 7:**
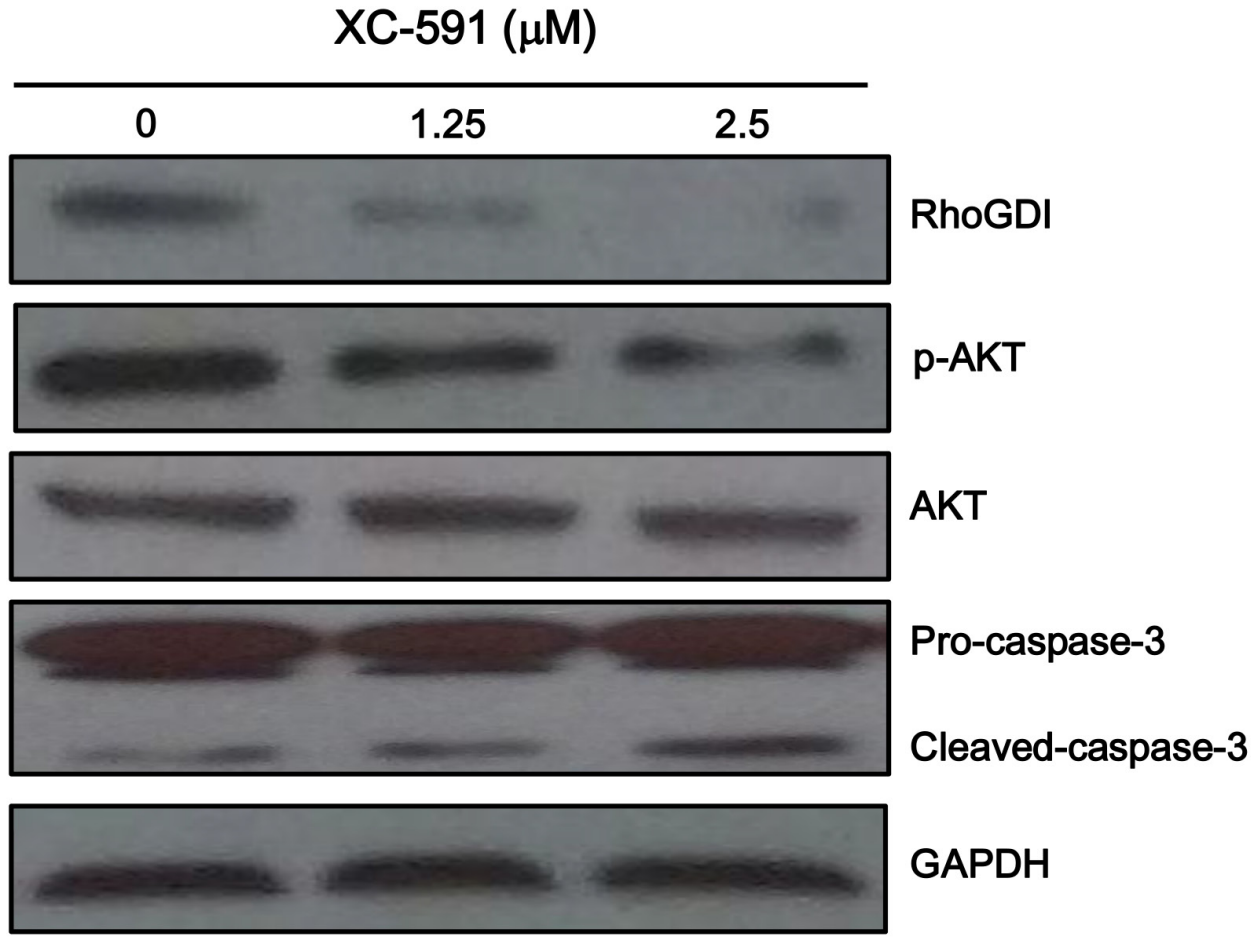
XC-591 inhibited RhoGDI, activated caspase-3 and reduced phosphorylated Akt 4T1 cells were treated with XC-591 at various concentrations for 48 h and then were analyzed the expression levels of RhoGDI, p-AKT, AKT, pro-caspase-3 and cleaved caspase-3 by western blot.

The predicted drug target protein of XC-591 is Rho GDP-dissociation inhibitor 1 (RhoGDI). The protein expression level of RhoGDI was examined by western blot after treatment with XC-591. As shown in Figure 7, RhoGDI was inhibited obviously in the XC-591 treated 4T1 cells.

We investigated whether Akt was involved in XC-591-mediated proliferation inhibitory effect. Our result showed XC-591 clearly decreased phosphorylated Akt (Figure 7). Furthermore, we attempted to study the impact of XC-591 on the activation of caspase-3. XC-591 treatment contributed to an obvious increase of cleaved caspase-3 (Figure 7).

## DISCUSSION

Because an obvious improvement of cancer molecular biology has been achieved recently, novel small molecule targeted drugs, which target key proteins of oncogenic pathways, have shown good effects in the management of many cancers [7–9].

In this study, we attempted to study the biological activities of XC-591, a new benzothiazole-2-thiol derivative, in detail. XC-591 showed obvious anti-proliferative activity on many cancer cells by MTT assay. Compared with other tumor cells, murine mammary tumor cell line 4T1 was more sensitive to XC-591 than others and thus was chosen for further study. XC-591 administered p.o. displayed a marked antitumor activity in 4T1 tumor models. The histological analysis showed XC-591 significantly reduced percentages of PCNA-positive nuclei, increased percentages of TUNEL positive nuclei and inhibited angiogenesis in a concentration-dependent way. During the whole experiment, no potential toxicity induced by XC-591 treatment was observed. In addition, in order to further study the molecular mechanism, we used western blot to examine the expression level of RhoGDI (predicted drug target protein), cleaved caspase-3 and phosphorylated Akt. The data showed that XC-591 could inhibit RhoGDI, activate caspase-3 and reduce phosphorylated Akt.

Rho family GTPases work in diverse cellular functions, including morphology, migration, gene transcription and cell cycle [10]. RhoGDI can inhibit nucleotide exchange and membrane association to down-regulate activities of Rho family GTPases [11]. RhoGDI is overexpressed in diverse human cancers, including lung cancer, melanoma, ovarian cancer and breast cancer [12–14]. The increased RhoGDI is associated with radiochemotherapy resistance and poor progonosis [15]. For example, a comparative proteomic study showed that RhoGDI was increased in oral squamous cell carcinoma and validated as an independent prognostic factor [16]. Furthermore, RhoGDI was identified as a metastasis-associated protein in colon and prostate cancer [17]. In addition, it has been reported that RhoGDI could stimulate the transcriptional activity of estrogen receptor α(ERα) via a RhoGTPase-dependent pathway that acts on estrogen receptor co-activators GRIP1 and CBP/p300 in breast cancer [18]. RhoGDI1 could increase both ligand-dependent and -independent ERα activity in breast cancer [19]. Thus, RhoGDI is a very promising anti-cancer target protein in breast cancer.

The important role of RhoGDI in tumor growth is supported by a number of important experimental observations. The suppression of RhoGDI alone by siRNA has a profound inhibitory effect on the growth of hepatocellular carcinoma [20]. Overexpressed RhoGDI can inhibit the induction of apoptosis by cytotoxic drugs in breast cancer cells. Silencing of RhoGDI by siRNA can increase the sensitivity of chemotherapy drugs, such as etoposide, doxorubicin and so on [21, 22]. These findings mentioned above showed that it is a good method to treat cancer by down-regulating RhoGDI. However, until now, no specific RhoGDI small molecule inhibitors have been designed and studied.

In conclusion, we developed a new benzothiazole-2-thiol derivative called XC-591. It showed good anti-cancer activity without obvious toxicity *in vitro* and *in vivo*. The molecular mechanism study showed that XC-591 could inhibit its target protein RhoGDI, and thus activated caspase-3 and decreased phosphorylated Akt. The present data may be useful for further exploration of this new small-molecule inhibitor in the treatment of breast cancer.

## MATERIALS AND METHODS

### Cell culture

Mouse colon carcinoma cell line CT26, mouse mammary tumor cell line 4T1, mouse Lewis lung carcinoma cell line LLC, mouse melanoma cell line B16, mouse fibrosarcoma cell line MethA, human lung carcinoma cell line A549, human cervical cancer cell line HeLa, human prostate cancer cell line DU145, mouse mammary normal epithelial cell line MM3MG and human normal kidney cell line HK2 were obtained from American Type Culture Collection (ATCC, Manassas, VA, USA). Human normal liver cell line LO2 was purchased from Cell biology of shanghai institute, shanghai, china. They were cultured in DMEM (Life Technologies, Bedford, MA, USA) or RPMI 1640 (Life Technologies).

### Synthesis of XC-591

The route adapted for the synthesis of compound XC-591 was reported before [6].

### MTT assay

Cells were seeded in 96-well plates and cultured for 24 h, followed by XC-591 treatment for 48 h. According to the instructions, MTT was done as reported before [6].

### Colony formation assay

4T1 cells were plated and then treated with 0, 1.25 and 2.5μM of XC-591, respectively. After 7-10 days of incubation, the cells were stained with 0.5% crystal violet in absolute ethanol and colonies were counted under dissection microscope.

### *In vivo* tumor experiment

To study the antitumor activities of XC-591 *in vivo*, 4T1 mice mammary tumor metastatic models were established. In brief, 1 × 10^5^ 4T1 cells were subcutaneously injected into the right dorsal flank of 6 to 8 weeks old female BALB/c mice. The tumor-bearing mice were randomly put into the following three groups (ten mice per treatment group) and each mouse received the corresponding treatment by intragastric administration once daily: (a) control group; (b) XC-591, 50 mg/kg; (c) XC-591, 100 mg/kg. Tumor volumes were evaluated according to the following formula: tumor volume (mm^3^) = 0.52 × length × width^2^. After sacrificed, tumor net weight was weighed. Autopsy was performed to assess the number and diameter of the metastatic nodules of lung.

### Immunohistochemistry

The primary antibody for PCNA was purchased from Santa Cruz Biotechnology (sc-7907, Santa Cruz, CA, USA). The primary antibody for CD31 was purchased from Abcam (ab28364, Abcam, Cambridge, United Kingdom). According to instructions of the Envision System-HRP method (DakoCytomation Inc, Carpinteria, CA, USA), tumor sections were stained.

### TUNEL assay

According to the instructions, TUNEL (terminal deoxynucleotidyl transferase mediated dUTP nick-end labeling) was completed to assess the percentage of apoptotic cells within tumors. Percent apoptosis was determined by counting the number of apoptotic cells and dividing by the total number of cells in the field (5 high power fields/slide).

### Toxicity assessment

To study the potential side effects in the XC-591 treated mice, they were continuously observed for relevant indexes such as weight loss, ruffled fur, diarrhea, anorexia, skin ulcer or toxic deaths. After sacrificed, various organs (lung, liver, kidney, heart, and spleen) were stained with H&E, and observed by two pathologists in a blinded manner. The levels of serum ALT and AST were determined with an automatic multifunction-biochemical analyzer. Complete blood counts and differentials were measured within each sample using an Abbott CELL-DYN 3700 hematology analyzer (Abbott Laboratories, Abbott Park, IL, USA).

### Western blot

The western blot was done as previously described [7]. The primary antibodies for RhoGDI and Akt/p-Akt were purchased from Cell Signaling Technology (Beverly, MA, USA). The primary antibodies for caspase-3 and GAPDH were acquired from Santa Cruz Biotechnology.

### Statistical analysis

Statistical analysis of the differences in tumor volume, tumor net weight, animal weight, percentages of apoptosis, percentages of PCNA-positive nuclei and microvessel density were done using one-way analysis of variance (ANOVA). P< 0.05 was considered statistically significant. Statistical analysis of the differences in pulmonary metastasis was analyzed using the Mann-Whitney test. P < 0.05 was considered statistically significant.
